# Conditional diffusion-generated super-resolution for myocardial perfusion MRI

**DOI:** 10.3389/fcvm.2025.1499593

**Published:** 2025-01-24

**Authors:** Changyu Sun, Neha Goyal, Yu Wang, Darla L. Tharp, Senthil Kumar, Talissa A. Altes

**Affiliations:** ^1^ Department of Chemical and Biomedical Engineering, University of Missouri, Columbia, MO, United States; ^2^ Department of Radiology, University of Missouri, Columbia, MO, United States; ^3^ Department of Medicine, University of Missouri, Columbia, MO, United States; ^4^ Department of Biomedical Sciences, University of Missouri, Columbia, MO, United States

**Keywords:** super-resolution, myocardial perfusion MRI, deep learning, diffusion probabilistic models (DDPM), conditional generative model, dynamic contrast-enhanced MRI (DCE MRI)

## Abstract

**Introduction:**

Myocardial perfusion MRI is important for diagnosing coronary artery disease, but current clinical methods face challenges in balancing spatial resolution, temporal resolution, and slice coverage. Achieving broader slice coverage and higher temporal resolution is essential for accurately detecting abnormalities across different slice locations but remains difficult due to constraints in acquisition speed and heart rate variability. While techniques like parallel imaging and compressed sensing have significantly advanced perfusion imaging, they still suffer from noise amplification, residual artifacts, and potential temporal blurring due to the rapid transit of dynamic contrast vs. the temporal constraints of the reconstruction.

**Methods:**

This study introduces a conditional diffusion-based generative model for myocardial perfusion MRI super resolution, addressing the trade-offs between spatiotemporal resolution and slice coverage. We adapted Denoising Diffusion Probabilistic Models (DDPM) to enhance low-resolution perfusion images into high-resolution outputs without requiring temporal regularization. The forward diffusion process introduces Gaussian noise incrementally, while the reverse process employs a U-Net architecture to progressively denoise the images, conditioned on the low-resolution input image.

**Results:**

We trained and validated the model on a retrospective dataset of dynamic contrast-enhanced (DCE) perfusion MRI, consisting of both stress and rest images from 47 patients with heart disease. Our results showed significant image quality improvements, with a 5.1% reduction in nRMSE, a 1.1% increase in PSNR, and a 2.2% boost in SSIM compared to GAN-based super-resolution method (*P* < 0.05 for all metrics using paired *t*-test) in retrospective study. For the 9 prospective subjects, we achieved a total nominal acceleration of 8.5-fold across 5–6 slices through a combination of low-resolution acquisition and GRAPPA. PerfGen outperformed GAN-based approach in sharpness (4.36 ± 0.38 vs. 4.89 ± 0.22) and overall image quality (4.14 ± 0.28 vs. 4.89 ± 0.22), as assessed by two experts in a blinded evaluation (*P* < 0.05) in prospective study.

**Discussion:**

This work demonstrates the capability of diffusion-based generative models in generating high-resolution myocardial perfusion MRI from conditional low-resolution images. This approach has shown the potentials to accelerate myocardial perfusion MRI while enhancing slice coverage and temporal resolution, offering a promising alternative to existing methods.

## 1 Introduction

Improving myocardial perfusion MRI is critical for assessing perfusion defects, requiring a balance between high spatial resolution, temporal fidelity, and slice coverage (1–3). Clinically, sufficient spatial resolution is necessary to detect subtle perfusion abnormalities but achieving enough spatial resolution (<3.0 mm) (4) and extensive slice coverage is particularly challenging under high heart rate conditions. The need to capture more slices (≥3 slices) within a short acquisition window further complicates the ability to fully resolve both motion and perfusion dynamics (5).

Recent techniques such as parallel imaging and compressed sensing (6–8), using both Cartesian (2) and non-Cartesian sampling (7, 9), have made progress in accelerating acquisition and increasing resolution in myocardial perfusion MRI. However, there remains open questions regarding the trade-offs between spatial and temporal fidelity, motion correction (10), as well as the potential for residual artifacts (11). Moreover, these methods typically require the complete acquisition of the entire temporal series to apply temporal regularization (1, 2) and motion correction, which can hinder the ability to display real-time images during contrast inflow and washout.

Given the ongoing challenges, there remains a need for alternative strategies to increase imaging speed for high temporal resolution and expanded slice coverage while simultaneously maintaining spatiotemporal fidelity. Low-resolution (LR) acquisitions inherently allow for faster imaging and higher signal-to-noise ratio (SNR), which can be crucial for capturing rapid contrast changes and minimizing the effects of cardiac and respiratory motion. By leveraging super-resolution (SR) methods (12, 13), these images can be enhanced to achieve higher spatial fidelity, offering a balance between imaging speed and diagnostic quality.

However, low-resolution perfusion may suffer from reduced spatial details and fidelity, as well as more severe dark rim artifacts and partial volume effects (14). These artifacts can interfere with accurate perfusion analysis and affect diagnostic outcomes. To address this, strategies must be developed to compensate for the loss of spatial resolution and mitigate artifacts. Recent advances in deep learning, particularly generative models, provide a promising way for enhancing the quality of LR images in cardiac MR imaging (15–18). However, Generative Adversarial Networks (GAN) are prone to experience unstable training and mode collapse issues (19). In contrast, diffusion models have proven to produce high-quality images with robust training stability and superior image quality (12, 20–21). Additionally, diffusion generative models offer a robust mechanism for improving spatial resolution of myocardial perfusion MRI without relying on temporal regularization. Previous studies have investigated GAN-based generative models on cardiac MRI (15, 18), but applying generative models to myocardial perfusion MRI has not been explored. By conditioning the diffusion model on low-resolution perfusion images, it is possible to enhance image detail while retaining the benefits of rapid acquisition, high temporal resolution and expanded slice coverage.

We propose to develop a conditional diffusion-based generative model for myocardial perfusion MRI super resolution, termed PerfGen, that leverages existing clinical imaging protocols and data to generate myocardial perfusion images conditioned by low-resolution images. This study explores the proof-of-the-concept that diffusion generative models can be integrated with myocardial perfusion MRI to synthesize high-resolution (HR) perfusion images and demonstrated its feasibility to accelerate the acquisition. This model provides an alternative solution that balances spatial resolution, temporal fidelity, and slice coverage, offering a new way for efficient and high-quality myocardial perfusion MRI.

## 2 Materials and methods

### 2.1 Data acquisition and preprocessing

All patients provided informed consent, and all studies were performed in accordance with protocols approved by our institutional review board.

#### 2.1.1 Retrospective myocardial perfusion data

Dynamic contrast-enhanced (DCE) perfusion data were collected from 47 heart disease patients using standard clinical MRI protocols at the University of Missouri-Columbia Hospital. The dataset was divided into an 80:20 split, with 38 patients for training and 9 patients for testing. Each subject had 3 short-axis slices (base, mid, and apex), with all temporal frames used, resulting in a total of 8,040 images for training and 1,830 images for testing. A mixed dataset with both rest and stress perfusion data were collected using gadolinium contrast for perfusion and Regadenoson for stress with free breathing acquisition. Prospective electrocardiogram triggering was used for all patients. Within the training group, 15 patients underwent rest perfusion only, and 23 underwent stress perfusion only. For the testing group, 3 subjects were assessed under rest conditions and 6 under stress. All testing data and most training data were acquired on a 1.5 T MAGNETOM Aera (Siemens Healthineers), except for 4 subjects from the training group were imaged using a 3 T MAGNETOM Vida (Siemens Healthineers).

Imaging parameters for the gradient echo perfusion sequence included a repetition time of 2.2–2.3 ms, echo time of 1.08 ms, flip angles between 12° and 15°, resolution of 2.3–2.4 mm × 2.3–2.4 mm, 60–80 temporal measurements, and a GRAPPA acceleration rate of 2. We use chest and spine phased-array receiver coils (20–34 channels) with an acquisition matrix of 160 × 120–160, a temporal resolution of 138–184 ms per slice, a saturation pulse delay of 100–120 ms, and acquire 3 slices per R-wave peak to R-wave peak (RR) interval.

#### 2.1.2 Prospective myocardial perfusion data

Nine DCE rest perfusion patient data were collected at University of Missouri-Columbia Hospital using a 3 T MAGNETOM Vida (Siemens Healthineers), with 8 acquired using GRAPPA-3 and 1 using GRAPPA-2. Prospective electrocardiogram triggering was used for all patients. Five to six short-axis myocardial perfusion slices were acquired per RR interval during free breathing. Imaging parameters of the gradient echo perfusion sequence included a repetition time of 2.2–2.3 ms, echo time of 1.08 ms, flip angles between 12° and 15°, resolution of 2.3–2.4 mm × 2.3–2.4 mm, 60 temporal measurements, and a GRAPPA acceleration rate of 2–3. Late gadolinium enhancement (LGE) imaging was used as a reference for validating the super-resolved perfusion defects, particularly in the presence of late enhanced regions. The acquisition matrix size is 160 × 48–62 following a 35%–36% low-resolution acceleration, with 16–22 actual phase encoding lines acquired using GRAPPA-3. The temporal resolution was 36.8–50.6 ms per slice, with an inversion time of 100 ms for the saturation pulse, resulting in a total acquisition time of 118.4–125.3 ms per slice. For the GRAPPA-2 data, the acquisition matrix size is 176 × 62 following a 35% low-resolution acceleration, with 32 actual phase encoding lines acquired using GRAPPA-2. The temporal solution was 73.6 ms per slice, resulting in a total acquisition time of 136.8 ms.

#### 2.1.3 Low-resolution data preparation

**Figure 1:**
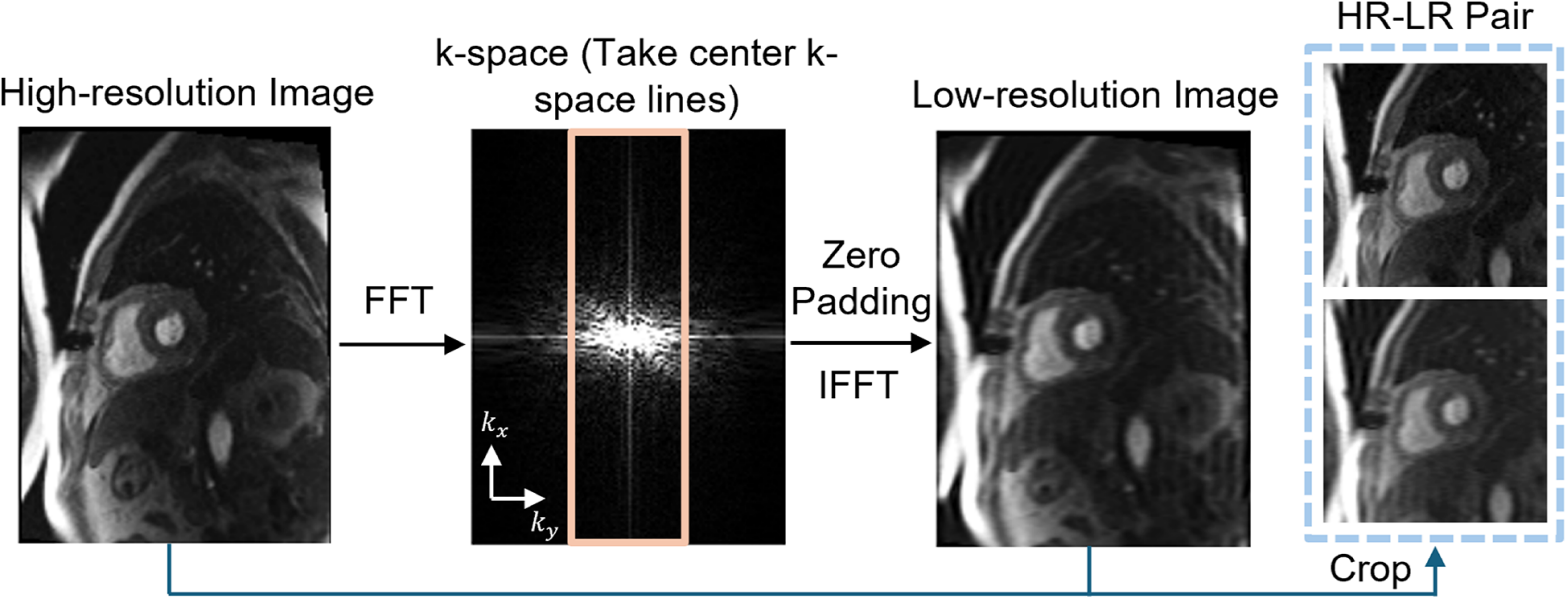
Illustration of the framework of synthetic data pipeline. High-resolution (HR) myocardial perfusion images are processed to generate synthetic low-resolution (LR) images. This involves Fast Fourier transform (FFT) of the perfusion image, taking the center k-space lines, applying zero-padding, and performing an inverse FFT (IFFT), resulting in paired HR and LR images.

To simulate LR perfusion data from HR perfusion images for model training, we used the following steps to generate LR and HR pairs (Figure 1). Fast Fourier Transform was applied to the original HR magnitude image to convert to k-space domain for retrospective experiments. The center 30%–50% of the phase-encoding lines were used, with the dynamic low-resolution ratio aiming for data augmentation. The outer k-space lines were zero-padded with the data in the readout maintained and converted back to the image domain using an inverse fast Fourier transform followed by taking the absolute value. Both the synthesized LR images and paired HR images were cropped to the same central 96 × 96 matrix size followed by the image normalization. The cropping was performed manually to position the heart within the cropped region. On-the-fly data augmentation included random vertical flip and horizontal flip, each applied with a probability of 50%. HR perfusion images served as the reference, and the LR synthetic images were enhanced using zero-padding and PerfGen. In the prospective study, the multi-coil complex-valued k-space data was truncated by setting the phase resolution to 35% in the sequence.

### 2.2 Conditional generation with denoising diffusion probabilistic models

Given a dataset of LR-HR perfusion MRI pairs, $D=(x_{i,}\;y_{i}{\}}_{i=1}^{N}$, which are samples from an unknown conditional distribution of high-resolution myocardial perfusion MRI domain, a parametric approximation of p(y|x) was learned through a stochastic iterative refinement process that maps the source LR image *x* to target HR image $\hat{y_{0}}$. We adapted the Denoising Diffusion Probabilistic Models (DDPM) and Image Super-Resolution via Iterative Refinement (SR3) (12) to generate HR MR perfusion images from LR image through diffusion process.

**Figure 2:**
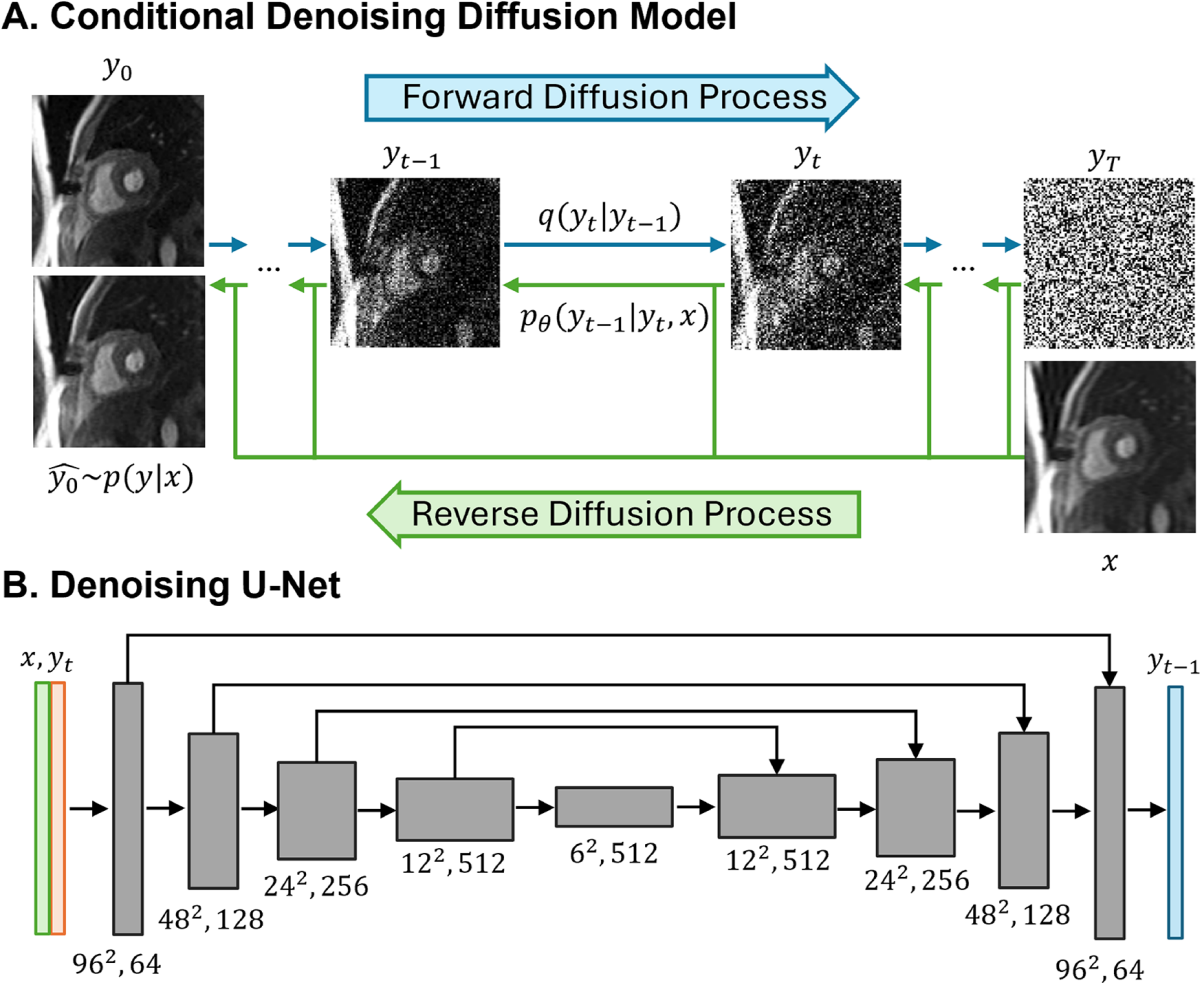
Illustrations of the conditional denoising diffusion model and the denoising U-Net architecture. **(A)** The forward diffusion process *q* (left to right) gradually add noise to the high-resolution image *y* over *T* steps until it converges to pure Gaussian noise *y*. The reverse diffusion process *p* (right to left) iteratively denoises the noisy images, conditioned on the low-resolution (LR) image *x*, to recover the high-resolution image. **(B)** The input to the U-Net is composed of two channels: the LR image *x* concatenated with the noisy image *y* at timestep *t*. The model outputs the denoised image *y*.

Figure 2A provides an illustration of a conditional diffusion-based model to map Gaussian noise $y_{T}$ to a HR image $\hat{y_{0}}$, conditioned on the source LR image *x*. The forward diffusion process *q* follows the Markov process to gradually add Gaussian noise to the HR perfusion image $y_{0}$ step by step until the image converges to a pure Gaussian distribution $y_{T}$. The reverse process *p* utilizes a U-Net model (22), trained to conditionally denoise the image to reconstruct a HR perfusion image $\hat{y_{0}}$ using the LR perfusion image *x* as the guidance.

#### 2.2.1 Forward diffusion process

The forward diffusion process gradually added Gaussian noise to the HR perfusion image $y_{0}$ over T iterations until the image converges to a Gaussian distribution via the diffusion kernel (Equation 1). Equation 2 provides the complete generation process.

(1) $$q(y_{t}\;|\;y_{t-1})=N(y_{t}\;|\;\sqrt{\alpha_{t}}y_{t-1},(1-\alpha_{t})I),$$

(2) $$q(y_{1:T}\;|\;y_{0})=\prod_{t=1}^{T}\;q(y_{t}\;|\;y_{t-1}),$$

where $\alpha_{t},\;1\leq t\leq T$ are variance schedule subject to $0<\alpha_{t}<1$, *I* is the identity matrix. T is set to 2,000, and the added Gaussian noise to the HR image generated a sequence of noisy images with increasing noise level $y\in [y_{1},y_{2},\ldots ,y_{T}]$.

Specifically, $q(y_{t})$ can be obtained directly from $y_{0}$ at any time step without iterations where $\gamma_{t}=\prod_{i=1}^{t}\;\alpha_{t}$ (Equation 3).

(3) $$q(y_{t}\;|\;y_{0})=N(y_{t}\;|\;\sqrt{\gamma_{t}}y_{0},(1-\gamma_{t})I),$$

#### 2.2.2 Reverse Diffusion Process

The reverse diffusion process is defined as a reverse Markov process, starting from Gaussian noise $y_{T}$ and progressively denoised to reconstruct the HR perfusion images $\hat{y_{0}}$ (Equations 4–6):

(4) $$p_{\theta }(y_{0:T}\;|\;x)=p(y_{t})\;\prod_{t=1}^{T}\;p_{\theta }(y_{t-1}\;|\;y_{t},x),$$

(5) $$p(y_{T})=N(y_{T}\;|\;0,\;I),$$

(6) $$p_{\theta }(y_{t-1}\;|\;y_{t},\;x)=N(y_{t-1}\;|\;\mu_{\theta }(x,y_{t},\gamma_{t}),\sigma_{t}^{2}I)$$

$p_{\theta }(y_{t-1}\;|\;y_{t},\;x)$ is the posterior distribution to be learned, distribution variance $\sigma_{t}^{2}$ is fixed to be $1-\alpha_{t}$, distribution mean $\mu_{\theta }(x,y_{t},\gamma_{t})$ is reparametrized as (Equation 7):

(7) $$\mu_{\theta }(x,y_{t},\alpha_{t})=\frac{1}{\sqrt{\alpha_{t}}}\left[y_{t}-\frac{1-\alpha_{t}}{\sqrt{1-\gamma_{t}}}f_{\theta }\;(x,y_{t},\gamma_{t})\right]$$

where $f_{\theta }(x,y_{t},\gamma_{t})$ is the denoising model which takes the source LR perfusion image *x* and a noisy image $y_{t}$ to predict the noise ϵ.

After the parametrization, each denoising step in the reverse process will be (Equation 8):

(8) $$y_{t-1}\leftarrow \frac{1}{\sqrt{\alpha_{t}}}\left[y_{t}-\frac{1-\alpha_{t}}{\sqrt{1-\gamma_{t}}}f_{\theta }(x,y_{t},\gamma_{t})\right]+\sqrt{1-\alpha_{t}}\epsilon_{t}$$

where $f_{\theta }(x,y_{t},\gamma_{t})$ is the denoising model, $\epsilon_{t}\;$ is the predicted noise at step t with $\epsilon_{t}∼N(0,I)$.

#### 2.2.3 Model implementation

We adapted the SR3-DDPM model to super-resolve a 2D low-resolution MR perfusion image into a HR image. The denoiser is achieved using a U-Net model and the optimization that employs KL-divergence to maximize the likelihood of the generated HR images $\hat{y_{0}}$ and the ground truth HR image $y_{0}$. L1-loss between the noise predicted by the network and the amount of noise added was used, and the objective function for training $f_{\theta }$ was defined as (Equation 9):

(9) $$\underset{\theta }{\mathrm{argmin}}\;L=\|f_{\theta }\left(x,\sqrt{\gamma }y_{0}+\sqrt{1-\gamma }\epsilon ,\gamma \right)-\epsilon {\|}_{1}^{1}$$

where $f_{\theta }$ represents the denoising U-Net model, *x* is the LR image, *y* is the corresponding HR image, ϵ is the added noise with ϵ∼N(0,I), γ is a scalar parameter related to the variance scheduler with $\gamma_{t}=\prod_{i=1}^{t}\;\alpha_{t}$.

The model starts with pure Gaussian noise and a LR perfusion image, using the corresponding HR perfusion image as the ground truth. The model will iteratively refine the noisy output through a U-Net model trained to denoise at various noise levels and generate images with the desired HR perfusion data distribution. By using the LR perfusion MR image to condition the generation process, the SR image is specifically determined to maintain anatomical consistency similar to the original LR perfusion images.

In the U-Net architecture (Figure 2B), the input comprised two channels representing the LR image and the noisy image, and one output channel, representing the generated less noisy HR images. The LR and HR pairs in our synthesis pipeline maintained the same matrix size, and the conditioning LR image was used at the shallowest level of the U-Net by channel-wise concatenating with the noisy image. Both the LR image and noisy image at time step t were encoded through a convolutional layer followed by two linear layers for further encoding. The U-Net structure was composed of convolution, group normalization, Swish activation, residual connections and pooling layers. The U-Net structure consisted of five levels, with the number of channels in each level being [64,128,256,512,512]. Each level contained two ResNet blocks (23) with a dropout rate of 0.2. At the bottleneck, an additional self-attention was applied after the convolution layers. The self-attention module employs convolutional layers to compute the query, key, and value representations for spatial attention. It is followed by another convolutional layer to refine the output and is interleaved with the original ResNet block at the bottleneck for enhanced feature representation. Detailed model architecture was depicted in Supplementary Figure S1.

PerfGen was implemented using Python and PyTorch on two 48GB NVIDIA A6000 GPUs. PerfGen had 92M trainable parameters. The model was trained for 50,000 iterations with AdamW optimizer with a learning rate of 3e-5 and a batch size of 128. During inference, we used DDPM sampling with full inference steps (T = 2,000).

### 2.3 Model evaluation

For synthetic data, to compare PerfGen with GAN-based super-resolution model trained on cardiac MRI (15), normalized Root-Mean-Square-Error (nRMSE), Peak Signal-to-Noise Ratio (PSNR), and Structural Similarity Index (SSIM) were calculated, using original HR images as reference. The metrics were evaluated within the 96 × 96 field of view, focusing specifically on the heart region. The nRMSE (24) was calculated as (Equation 10):

(10) $$nRMSE=\frac{1}{\max(I)-\min(I)}\sqrt{\frac{1}{N}\sum_{i=0}^{N-1}\;{\left({\tilde{I}}_{i}\;-I_{i}\right)}^{2}}$$

where $\tilde{I}\;$ is SR images super-revolved by PerfGen or GAN, *I* is the reference HR image, *N* is the total number of pixels, ${\tilde{I}}_{i}$ and $I_{i}$ are the pixel intensities at position *i* in the SR and HR images, respectively. PSNR (25) was calculated as (Equation 11):

(11) $$PSNR=10\times \log_{10}\;\left(\frac{255^{2}}{\frac{1}{N}\sum_{i=0}^{N-1}\;{\left({\tilde{I}}_{i}\;-I_{i}\right)}^{2}}\right)$$

SSIM (26) was calculated as (Equation 12):

(12) $$SSIM=\frac{\left(2\mu_{\tilde{I}}\;\mu_{I}+c_{1})(2\sigma_{\tilde{I,}I}+c_{2}\right)}{\left(\mu_{\tilde{I}}^{2}+\mu_{I}^{2}+c_{1})(\sigma_{\tilde{I}}^{2}+\sigma_{I}^{2}+c_{2}\right)}$$

where $\mu_{\tilde{I}}$ and $\mu_{I}$ are the average and variance of $\tilde{I}\;$ and I, $\sigma_{\;\tilde{I,}I}$ is the covariance of $\tilde{I}\;$ and I, and $c_{1}$ and $c_{2}$ are constants to prevent division by a near-zero denominator.

Differences between GAN super-resolved images and PerfGen super-resolved images were statistically tested using a paired *t*-test (*P* < 0.05). For prospective data, images super-resolved by two methods were qualitatively compared with LGE images at matched slice position to identify perfusion defects. One cardiologist and one radiologist scored prospectively acquired datasets on a 1–5 scale (1 being the worst and 5 being the best), assessing perfusion image sharpness and overall quality relative to clinical perfusion image standards. Differences between methods were assessed with the Wilcoxon signed-rank test.

## 3 Results

### 3.1 Model validation with synthetic data

#### 3.1.1 Qualitative comparison

**Figure 3:**
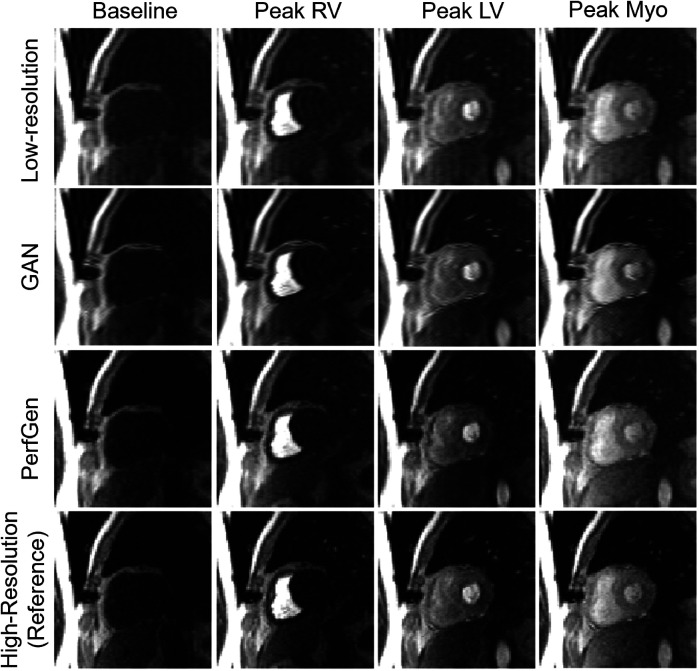
Comparison of myocardial perfusion images across perfusion phases for one retrospective test data. From left to right, the images show the perfusion image at baseline, peak right ventricle (RV), peak left ventricle (LV), and peak myocardium (Myo). Each column represents different time points, illustrating the progression of contrast perfusion through the myocardium, highlighting key cardiac phases with distinct contrast enhancement in the RV, LV, and myocardial tissue. Each row shows the low-resolution images, GAN-based super-resolved images, PerfGen super-resolved perfusion images, and high-resolution reference images.

Figure 3 compares the myocardial perfusion images across different phases of contrast enhancement during first-pass perfusion using synthetic test data. The PerfGen super-resolved images are compared to LR, GAN super-resolved and HR reference images at baseline, peak right ventricle (RV), peak left ventricle (LV), and peak myocardium (Myo). The results show an improvement in the image resolution and contrast for the PerfGen super-resolved images, allowing for enhanced visualization of contrast perfusion through the myocardium. The enhanced detail provided by PerfGen aligned better with the HR reference images than LR and GAN methods.

**Figure 4:**
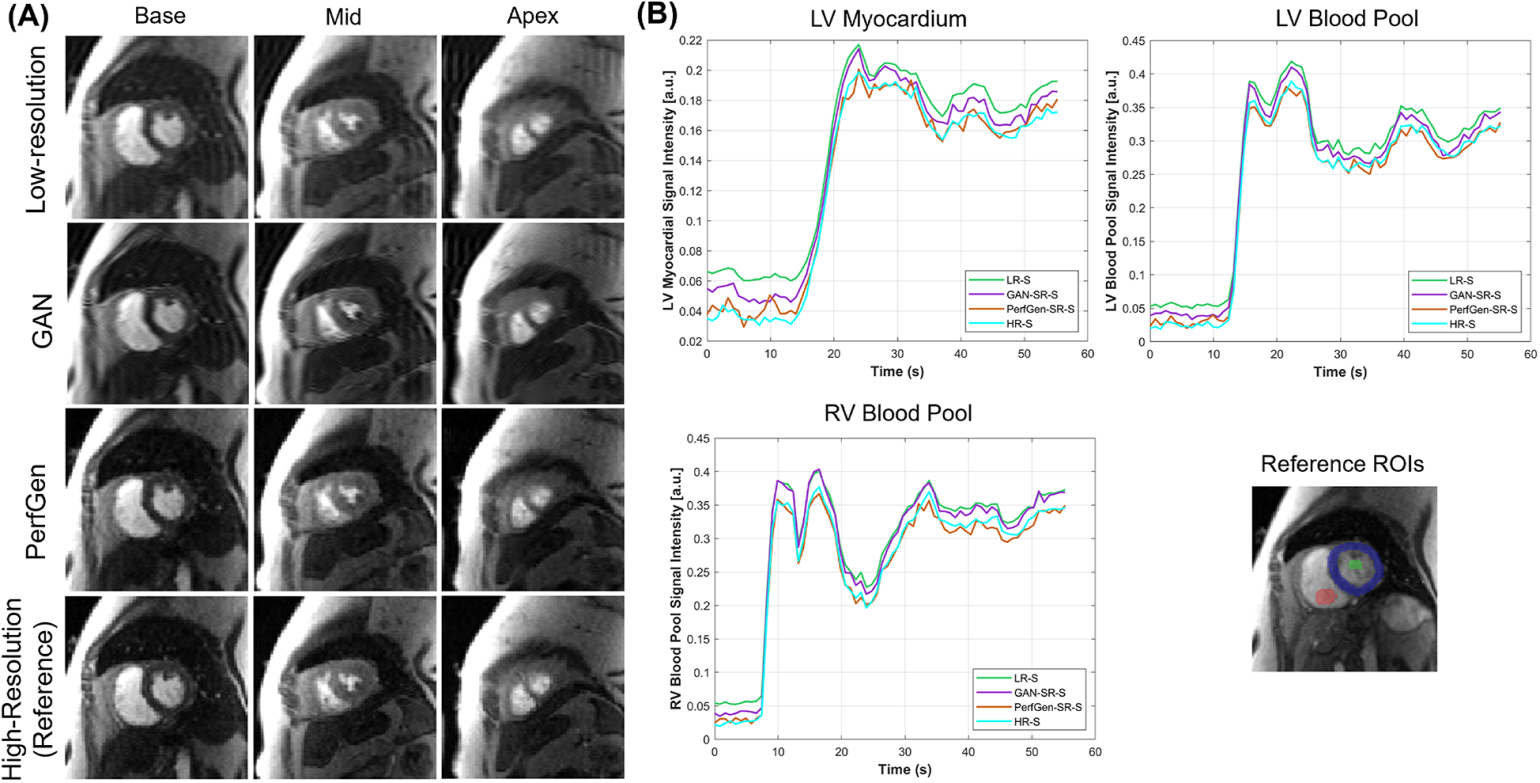
**(A)** Comparison of GAN-based super-resolved images, PerfGen super-resolved images and high-resolution (HR) perfusion images across different slice locations for one synthetic test data. From left to right, the myocardial perfusion images are shown at the base, midventricular, and apical slice locations. The top row showed low-resolution (LR) images, the following rows showed GAN-based and PerfGen super-resolved images, and the bottom row showed the corresponding HR reference images. This comparison highlights the effectiveness of PerfGen in enhancing image resolution and better alignment with reference images than GAN-based approach across various slice locations of the heart. **(B)** The signal-t plots illustrate the signal intensity of the basal slice in **(A)** in terms of the left-ventricular (LV) myocardial region, LV blood pool and right-ventricular (RV) blood pool changes over time. The spatially super-resolved images by PerfGen (PerfGen-SR-S) aligns better with the reference HR spatial images (HR-S) than the acquired LR spatial images (LR-S) and the GAN-based super-resolved images (GAN-SR-S).

Figure 4A further demonstrates the evaluation of the PerfGen by comparing myocardial perfusion images at the basal, midventricular, and apical slice locations. The PerfGen super-resolved images show enhanced resolution and structural details across all slice locations compared to the HR reference. Figure 4B shows the signal-t plot illustrating the changes in the LV myocardial region, LV blood pool, and RV blood pool. The spatially super-resolved images demonstrate better alignment with the reference high-resolution spatial images compared to the acquired LR spatial images and GAN-based super-resolved images.

**Figure 5:**
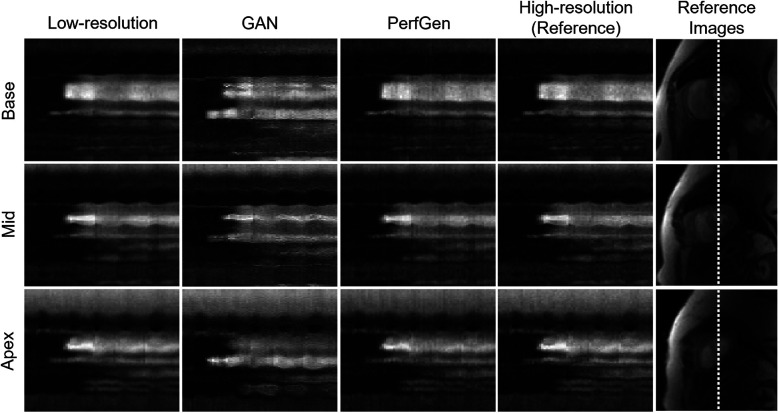
Comparison of cross-section profile along time plots corresponding to low-resolution (LR), GAN, PerfGen and reference high-resolution (HR) for the patient in Figure 4 were shown. PerfGen presents better temporal fidelity than zero-padded LR images and GAN-based super-resolved images compared to the reference HR x-t plots.

**Figure 6:**
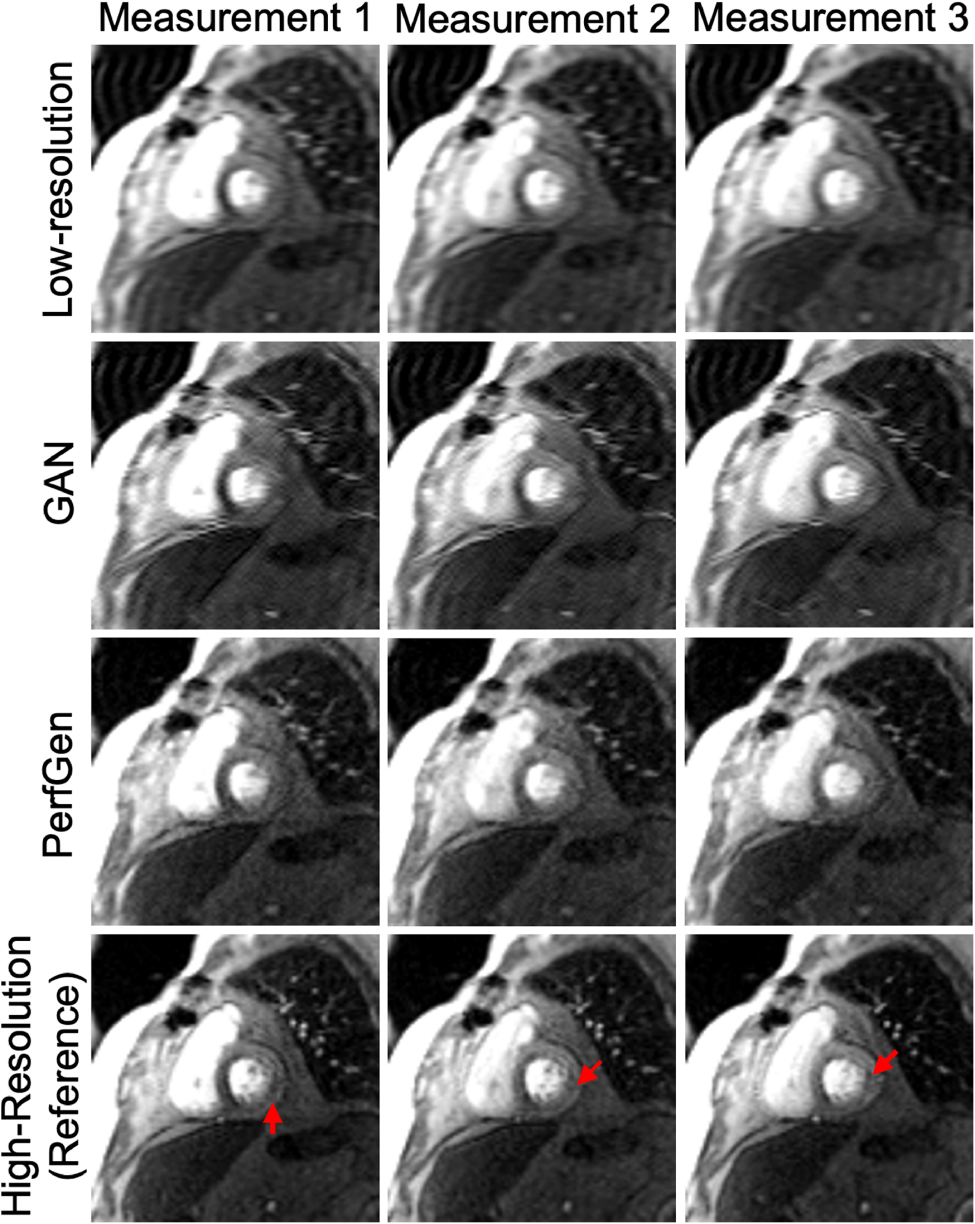
Example images from a retrospective patient undergoing regadenoson stress perfusion imaging with inducible myocardial ischemia with a moderate-sized defect in the basal septum and a small defect in the inferolateral wall. Low-resolution (LR) images, GAN-based super-resolution, PerfGen, and high-resolution (HR) images were compared as reference for image quality and their ability to illustrate perfusion defects. LR images, used as the baseline, appear blurred but indicates perfusion defects in the septum. The GAN-based super-resolution method improves image sharpness but shows slight mismatches with the HR reference in terms of image details in myocardial regions, with potentially reduced fidelity in depicting the small inferolateral wall perfusion defects (indicated by red arrows). PerfGen demonstrates greater visual similarity to the HR reference, more accurately highlighting perfusion defects in similar regions with higher fidelity.

Figure 5 presents example cross-sectional time-intensity profiles from the subject shown in Figure 4, comparing LR images, GAN super-resolved images, PerfGen super-resolved images, and reference HR images. The PerfGen super-resolved method shows better temporal fidelity than the LR images and GAN super-resolved images compared with the reference HR plots. Figure 6 shows the stress perfusion images of a patient with inducible myocardial ischemia. PerfGen demonstrates better alignment with HR reference images than LR images and GAN-based super-resolved images in terms of the overall image quality and the accurate perfusion defect detection.

#### 3.1.2 Quantitative comparison

**Figure 7:**
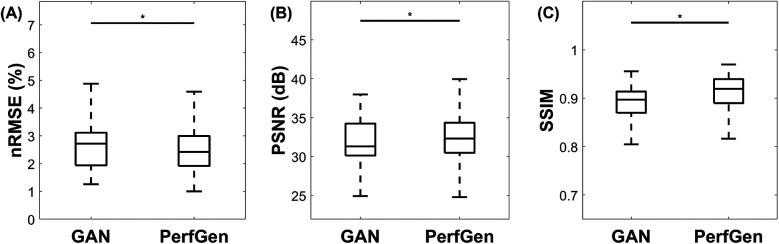
Quantitative comparison of GAN and PerfGen super-resolved myocardial perfusion images for nine tested datasets. Boxplots show (A) normalized root mean square error (nRMSE), (B) peak signal-to-noise ratio (PSNR), and (C) structural similarity index (SSIM) for GAN and PerfGen images. PerfGen demonstrates a significant improvement in all metrics, with lower nRMSE, higher PSNR, and higher SSIM compared to super-resolved images by GAN, with a reduction in nRMSE by approximately 5.1%, an increase in PSNR by 1.1%, and an improvement in SSIM by 2.2%. Statistical significance is indicated by the asterisks.

Figure 7 presents a quantitative comparison between GAN and PerfGen super-resolved myocardial perfusion images of nine testing datasets. The PerfGen method significantly outperformed the GAN-based approach across all evaluated metrics. Specifically, PerfGen achieved a 5.1% reduction in nRMSE (mean nRMSE: 2.68 ± 0.85% for GAN vs. 2.55 ± 0.84% for PerfGen, respectively), a 1.1% increase in PSNR (mean PSNR: 31.89 ± 2.82 dB for GAN vs. 32.24 ± 2.77 dB for PerfGen, respectively), and a 2.2% improvement in SSIM (mean SSIM: 0.87 ± 0.15 vs. 0.89 ± 0.16 for PerfGen, respectively). These improvements are statistically significant, as indicated by the asterisks in Figure 7, demonstrating the superior performance of PerfGen in enhancing image quality.

### 3.2 Model validation with prospectively acquired data

**Figure 8:**
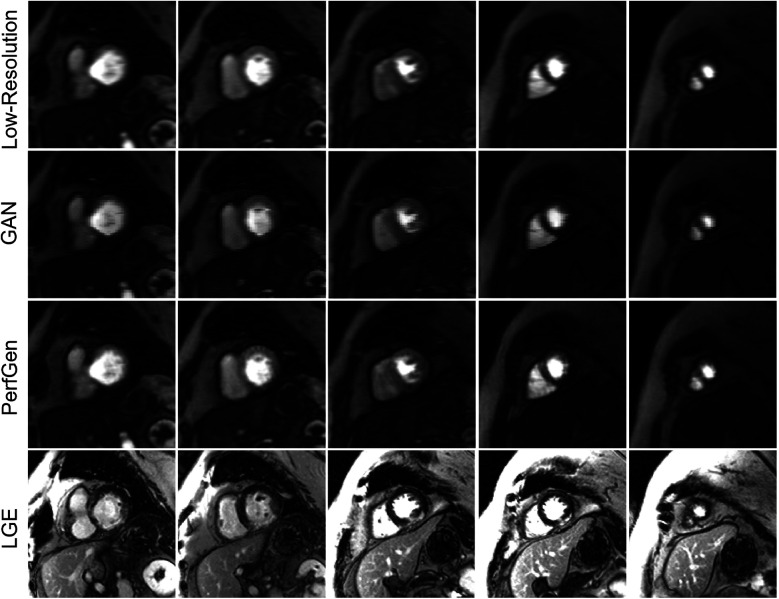
Comparison of low-resolution (LR), GAN super-resolved images, PerfGen super-resolved images and LGE images for one prospectively acquired myocardial perfusion dataset. The first row illustrates the LR perfusion images, followed by the super-resolved images by GAN and PerfGen. The last row shows the corresponding LGE images at similar slice locations. The PerfGen super-resolved perfusion images highlight perfusion defects that match the locations of defects observed in the LGE images. PerfGen demonstrates superior alignment with LGE compared to GAN-based super-resolved perfusion images, demonstrating the ability of PerfGen to recover and enhance important diagnostic features. This figure also shows how the combination of LR acquisition of 35% phase resolution and GRAPPA-2 can improve slice coverage, with five slices acquired and 2.86-fold higher temporal resolution for this patient.

Figure 8 compares the super-resolved perfusion images by PerfGen and GAN to both LR perfusion images and LGE images. The super-resolved images show perfusion defects that closely match the defects observed in the LGE images at corresponding slice locations, providing proof-of-concept that PerfGen can potentially identify the super-resolve perfusion defects from LR images. PerfGen demonstrates better alignment with LGE compared to GAN-based super-resolved perfusion images. Furthermore, the combination of a LR acquisition with 35% phase lines and GRAPPA-2 allowed the acquisition of five slices, with a 2.86-fold improvement in temporal resolution compared to clinical routine settings, demonstrating the improved image quality with improved slice coverage.

**Figure 9:**
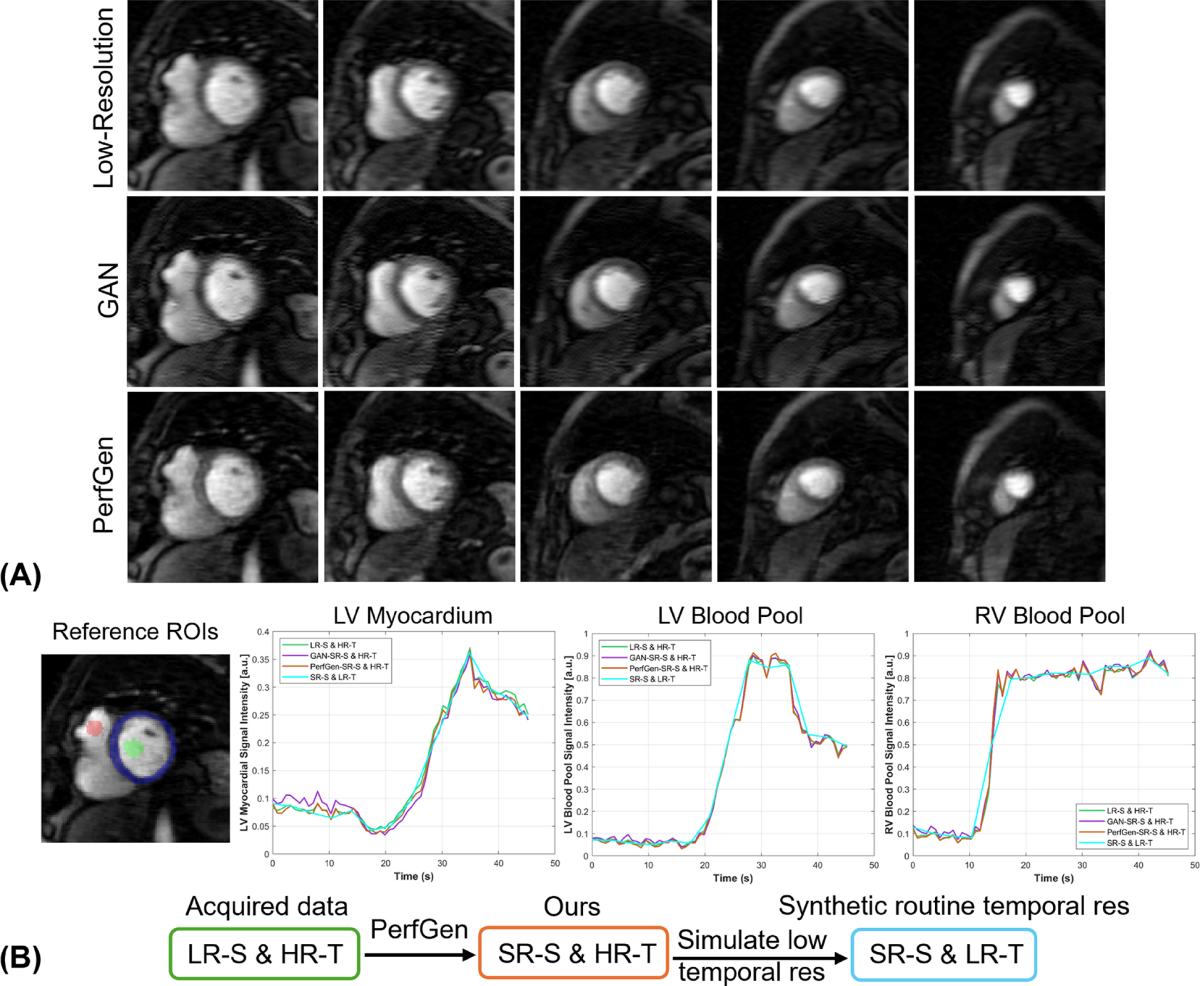
**(A)** Comparison of low-resolution (LR) perfusion images, GAN-based super-resolved images and PerfGen super-resolved images across different perfusion slices for one prospectively acquired test data. This figure shows the super-resolved images by PerfGen compared with low-resolution images using zero-padding and super-resolved images by GAN at different slice locations using GRAPPA-3 and LR of 35% phase resolution. PerfGen demonstrate improved contrast and details in the images than zero-padding images and GAN super-resolved images. **(B)** The signal-time plots show the intensity changes over time for the basal slice in **(A)** in terms of the LV myocardial region, LV blood pool, and RV blood pool. The acquired LR spatial image achieves 4.3-fold higher temporal resolution (green curve) compared to routine reference temporal resolution. PerfGen super-resolved spatial images maintain this high temporal resolution (orange curve) while capturing more detailed myocardial perfusion dynamics than zero-padding and GAN super-resolved images. The synthetic curve represents 4.3-fold lower temporal resolution, simulating a 2-fold accelerated acquisition. The higher temporal resolution (green, purple and orange curves) enables more accurate tracking of rapid perfusion changes compared to the smoother dynamics observed in the low temporal resolution curve (SR-S & LR-T blue curve) as supported by the comparison with low spatial resolution images (LR-S & HR-T, green curve). LV, left-ventricular; RV, right-ventricular; LR-S, low spatial resolution images; HR-T, high temporal resolution images; SR-S, super-resolved spatial images: low temporal resolution images.

Figure 9A provides evaluation of PerfGen in enhancing myocardial perfusion images across five different perfusion slices. Super-resolved images are compared with LR images using zero-padding for five prospectively acquired slice, showing improved contrast and image detail across various perfusion phases by PerfGen. The super-resolution method demonstrates improved visualization of image details that are not as obvious in the LR images or GAN super-resolved images acquired using GRAPPA-3 and 35% phase resolution. Figure 9B shows the signal-time plots of the intensity changes over time in the LV myocardial region, LV blood pool and RV blood pool. The plots demonstrate that achieving a 4.3-fold increase in temporal resolution and five slices covered in myocardial perfusion MRI, compared to the clinically used GRAPPA-2 and three slices, improves the ability to capture sharp transitions in contrast during myocardial perfusion as compared against a synthetic low temporal resolution curve using rate-2 acceleration. Supplementary Video 1 showed the movies of six slices from one prospective patient acquired with GRAPPA-3 and 35% phase encoding lines, comparing LR, GAN-based super-resolved and PerfGen super-resolved images.

**Figure 10:**
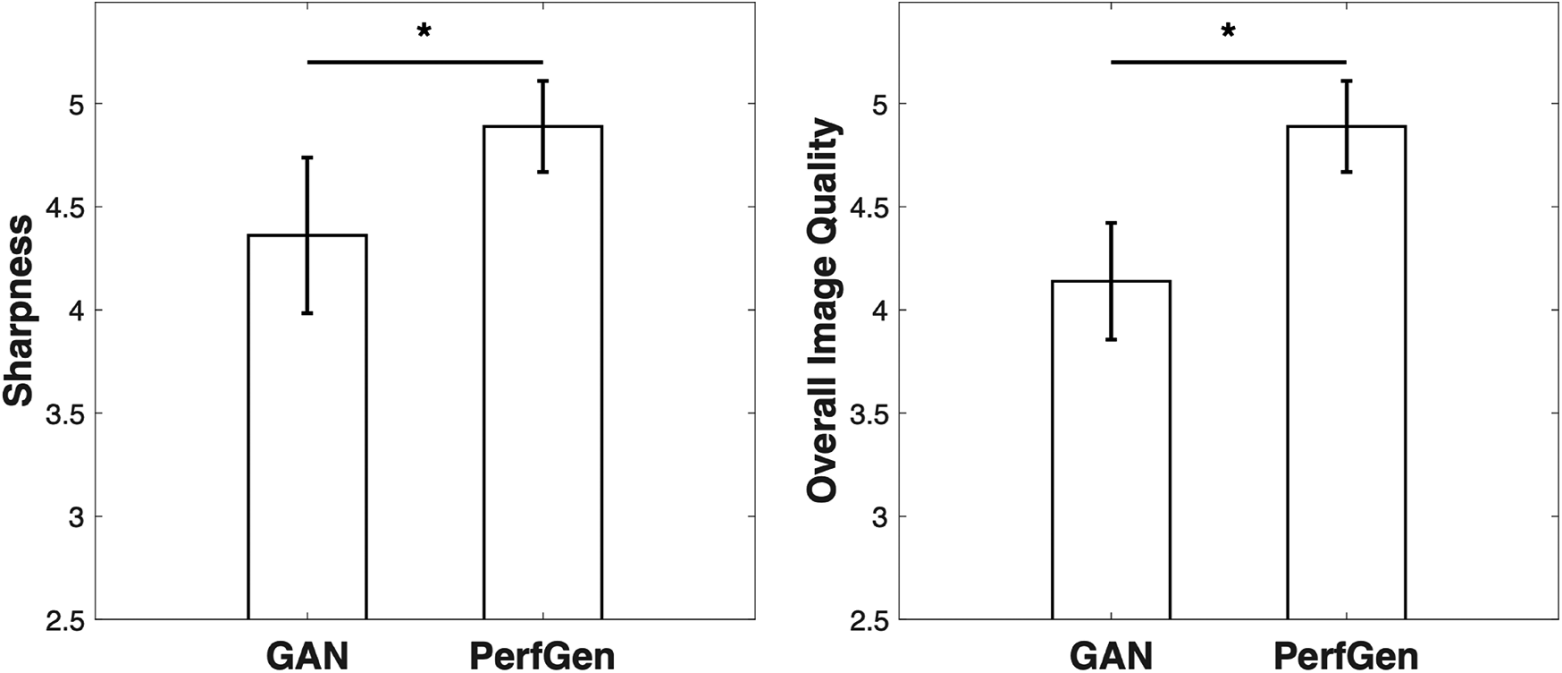
Quantitative comparisons of GAN and PerfGen methods for super-resolution of prospective perfusion images, evaluated on 9 prospective subjects and scored by two experts. PerfGen demonstrated significantly better performance than GAN in sharpness and overall image quality. Sharpness scores were 4.36 ± 0.38 for GAN and 4.89 ± 0.22 for PerfGen (*P* < 0.05), while overall image quality scores were 4.14 ± 0.28 for GAN and 4.89 ± 0.22 for PerfGen (*P* < 0.05).

For the nine prospectively acquired subjects, all slices were evaluated by two experts in Figure 10. For sharpness, the scores were 4.36 ± 0.38 for GAN and 4.89 ± 0.22 for PerfGen (*P* < 0.05); for overall image quality, the scores were 4.14 ± 0.28 for GAN and 4.89 ± 0.22 for PerfGen (*P* < 0.05).

PerfGen not only improves spatial resolution but also enhance critical image features, such as perfusion defects, that align well with LGE reference images, providing proof of concept for demonstrating the potential of super-resolution techniques in diagnostic accuracy in myocardial perfusion imaging.

## 4 Discussion

With the growing interest in myocardial perfusion MRI in identifying myocardial ischemia, there is an increased need for high spatiotemporal resolution and expanded slice coverage to accurately monitor dynamic changes in blood flow and myocardial perfusion. This poses a challenge in achieving acquisition speed to capture rapid change and fine details without sacrificing the quality and accuracy necessary for effective diagnosis. Low-resolution acquisition is an alternative approach that inherently allows for acceleration and higher SNR. However, the reduction in spatial and temporal details may degrade the image quality, influence the diagnosis accuracy and potentially impact the subsequent quantitative analysis.

In this study, we demonstrated that existing clinical perfusion MRI images can be effectively used to train a conditional diffusion generative model for super-resolution. We proposed a super-resolution pipeline that utilizes low-resolution myocardial perfusion MRI as the guidance after initial reconstruction by GRAPPA (27), which is also potentially applicable to compressed sensing (28) or unrolled network (29) outputs, offering a complementary approach to the existing workflows. When combined with GRAPPA (factor 2–3) in prospective acquisitions, this method offers a nominal 5.7–8.5 folds acceleration, allowing for better slice coverage and improved temporal resolution. This approach not only accelerates acquisition but also mitigates the loss of contrast and details typically associated with low-resolution imaging. We validated our model on an infarction patient using reference LGE images, with the perfusion defects showing consistent with the scar regions in LGE.

While diffusion generative models have demonstrated the training stability and high-quality image generation across various vision tasks (12, 20, 30), their application in generating myocardial perfusion MRI and integration with cardiac MRI have yet to be explored. The study demonstrates that the diffusion generative model produces myocardial perfusion images comparable to routine GRAPPA-2 perfusion images and outperform the compared GAN-based method, highlighting its potential to enhance temporal resolution and slice coverage for clinical use. This super-resolution approach provides several key advantages: (1) it effectively generates fine image details, outperforming one existing GAN-based super-resolution method, (2) the combination of low-resolution acquisition and GRAPPA reduces the risk of residual artifacts from highly accelerated undersampling (8.5-fold), (3) the GRAPPA-reconstruction by the vendor allows for real-time visualization for perfusion imaging, enabling real-time monitoring of contrast dynamics, and (4) the super-resolution process operates independently of the full temporal series, allowing for efficient image-by-image processing and minimize the potential loss of temporal fidelity.

Our results showed higher temporal resolution than the clinically used GRAPPA-2, where the higher temporal resolution enables better capture of fast perfusion dynamics. This enhancement can reduce temporal blurring, provide more precise time-intensity curves for quantitative analysis, and allow for more accurate assessment of myocardial ischemia. Additionally, higher temporal resolution can mitigate motion artifacts caused by cardiac and respiratory motion, resulting in images with better image quality and potentially more reliable diagnostic outcomes.

One of the potential limitations of this approach is that the super-resolved output cannot theoretically exceed the spatial resolution of the reference images used for training. A broader dataset would be beneficial for a more thorough model training. Although GRAPPA-reconstruction allows for real-time visualization, PerfGen requires additional time to enhance image quality and does not currently support real-time processing. While the diffusion generative model aims to learn the distribution of high-resolution perfusion images and generate high-quality outputs, and proof-of-concept studies highlight the promise of the PerfGen model, accurately detecting small perfusion defects remains challenging due to partial volume effects and dark rim artifacts. Addressing these potential limitations will require further training on larger datasets, optimized network architectures, and robust training strategies. Additionally, further studies are necessary to validate these findings in diverse clinical scenarios, particularly for assessing ischemic perfusion defects in stress perfusion MRI. It is important to note that the comparison between our method and GAN-based method involves a single diffusion generative model and one published GAN-based approach trained for cardiac MRI super-resolution which has been applied to various cardiac MRI applications (15, 31). This comparison is not intended as a comprehensive theoretical comparison between GANs vs. diffusion models. The relative performance of these models may also depend on factors such as the datasets used, the specific applications, and other implementation details. Currently, our findings are preliminary and serve as a proof-of-concept; additional clinical validation is necessary to assess the reliability of this approach. Future efforts could also explore integrating the super-resolution method with physics-guided self-supervised learning reconstruction networks for high-resolution perfusion MRI (32), and extend this approach with quantitative analysis (33–35). Further studies will be needed to establish the clinical utility and validate the diagnostic values of this method.

Overall, this work shows the capability of the conditional diffusion generative model in high-resolution myocardial perfusion MRI generation and demonstrates its feasibility to accelerate myocardial perfusion MRI acquisition, increase temporal resolution and slice coverage, and improve image quality without introducing significant artifacts or blurring.

## Data Availability

The raw data presented in this article are not readily available due to patient privacy. Requests to access the dataset should be directed to csyfc@missouri.edu.

## Supplementary material

Supplementary Table 1 Details of contrast agent and stress agent protocol

Supplementary Figure 1 Detailed depiction of the denoising U-Net architecture. The network takes in a two-channel input, where one channel represents the low-resolution condition image and the other contains the noisy image at time step t. The output is the denoised image at the subsequent time step t-1. The U-Net architecture consists of convolutional layers, group normalization, Swish activation, residual connections, and pooling layers. A convolution layer and two linear layers were used as the image encoder for the inputs. At the bottleneck, an additional self-attention layer was applied.

Supplementary Video 1 Comparison of zero-padded low-resolution, GAN-based super-resolved and PerfGen super-resolved myocardial perfusion images across six slices and perfusion measurements acquired with 35% phase encoding lines and GRAPPA-3. The first row shows the zero-padded low-resolution images, while the following rows present the corresponding GAN-based super-resolved images and PerfGen super-resolved images. The super-resolved images by PerfGen demonstrate improved spatial resolution and contrast, recovering key perfusion features that may be less apparent in the low-resolution images and GAN super-resolved images. This movie highlights the potential of PerfGen to enhance image quality and diagnostic accuracy for myocardial perfusion imaging.
