# Supplementary material for: Conditional diffusion-generated super-resolution for myocardial perfusion MRI
Source: Front Cardiovasc Med. 2025 Jan 24;12:1499593. doi: 10.3389/fcvm.2025.1499593 (PMC11802533; doi:10.3389/fcvm.2025.1499593)
Supplement: Supplementary Table 1 [file Table1.docx]

**Supplementary Table 1:** Details of contrast agent and stress agent protocol

| **Contrast Agent and Stressor Information** | **Administration Details** |
| --- | --- |
| Gadolinium Contrast Agent | Multihance |
| Contrast Dose (mmol/kg body weight) | 0.1 |
| Contrast Injection Rate (ml/s) | 3.5 |
| Saline Chasing Bolus (ml) | 20-30 |
| Saline Injection Rate (ml/s) | 3.5 |
| Stress Agent | Regadenoson |
| Stress Agent Dose (mg) | 0.4 |
| Aminophylline | Not Administered |

Note: Rest RR Interval (ms): 860.7 ± 153.5, measured for all patients with rest-only perfusion MRI in the current dataset. Stress RR Interval (ms): 745.5 ± 183.7, measured for all patients with stress-only perfusion MRI in the current dataset. No patients in this dataset have both rest and stress perfusion MRI. RR refers to the interval between successive R-waves in cardiac cycles.
