# Supplementary figures and images for: Conditional diffusion-generated super-resolution for myocardial perfusion MRI
Source: Front Cardiovasc Med. 2025 Jan 24;12:1499593. doi: 10.3389/fcvm.2025.1499593 (PMC11802533; doi:10.3389/fcvm.2025.1499593)

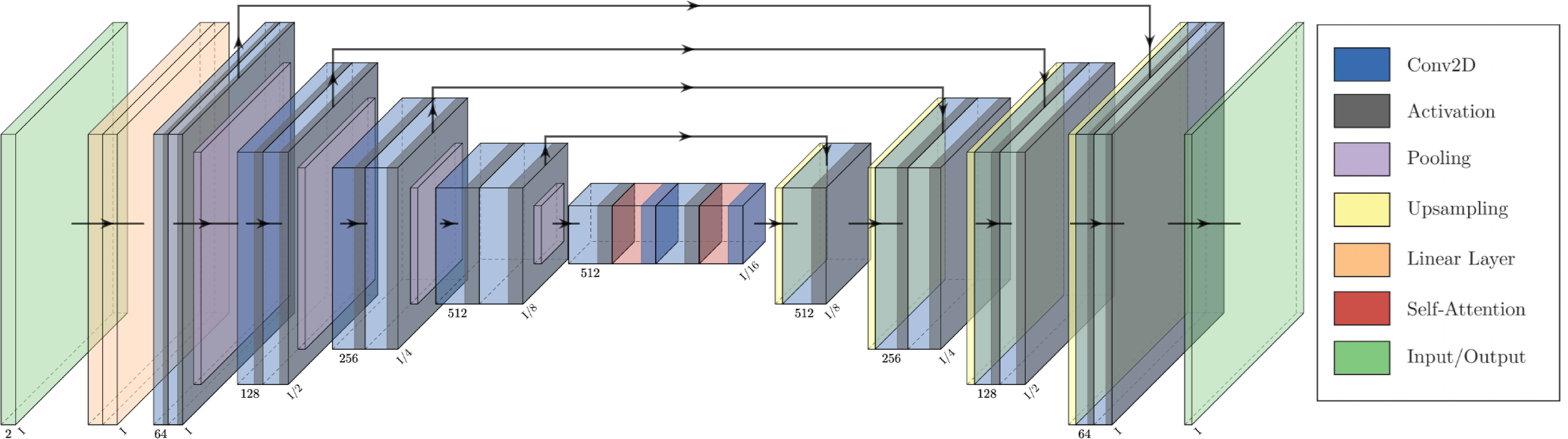

Supplement: Supplementary Figure 1 [file Image1.tiff]
